# Outcome of papillary versus clear cell renal cell carcinoma varies significantly in non-metastatic disease

**DOI:** 10.1371/journal.pone.0184173

**Published:** 2017-09-21

**Authors:** Nina Wagener, Dominic Edelmann, Axel Benner, Richard Zigeuner, Hendrik Borgmann, Ingmar Wolff, Laura M. Krabbe, Mireia Musquera, Paolo Dell’Oglio, Umberto Capitanio, Tobias Klatte, Luca Cindolo, Matthias May, Sabine D. Brookman-May, on behalf of the European Association of Urology (EAU) Young Academic Urologists (YAU) Kidney Cancer Group

**Affiliations:** 1 Department of Urology, Mannheim Medical Center, University of Heidelberg, Mannheim, Germany; 2 Division of Biostatistics, German Cancer Research Center, Heidelberg, Germany; 3 Department of Urology, Medical University of Graz, Graz, Austria; 4 Department of Urology, University Medical Center, University of Mainz, Mainz, Germany; 5 Department of Urology, Carl-Thiem-Klinikum Cottbus, Cottbus, Germany; 6 Department of Urology, University of Muenster Medical Center, Muenster, Germany; 7 Department of Urology, Hospital Clinic, University of Barcelona, Barcelona, Spain; 8 Unit of Urology and Division of Experimental Oncology, Urological Research Institute (URI), San Raffaele Scientific Institute, University Vita-Salute, Milan, Italy; 9 Department of Urology, Medical University of Vienna, Vienna, Austria; 10 Department of Urology, San Pio da Pietrelcina Hospital, Vasto, Italy; 11 Department of Urology, St. Elisabeth-Hospital Straubing, Straubing, Germany; 12 Department of Urology, Ludwig-Maximilians-University (LMU), Munich, Germany; National Institute of Health, UNITED STATES

## Abstract

Renal cell carcinoma (RCC) comprises a heterogenous group of tumors. Traditionally, papillary RCC (pRCC) is associated with a favorable outcome compared to clear cell RCC (ccRCC), while other series report equivalent or worse prognosis. In this paper we comparatively evaluate outcome of pRCC versus ccRCC in two large multi-institutional databases (cohort study), including distribution of pRCC subtypes 1 and 2. Retrospective data of 1,943 surgically treated pRCC patients from 17 European/ North American centers between 1984–2015 were compared to 5,600 ccRCC patients from a database comprising 11 European/ North American centers (1984–2011). Median follow-up was 64.6 months. Differences between pRCC, subtypes, and ccRCC were compared with t-tests, Chi^2-tests, and exact Fisher tests. Cancer-specific mortality was analyzed with cumulative incidence curves and Cox cause-specific hazard models. The robustness of our results was examined with sensitivity analyses. We present that cancer-specific mortality rates and variables as stage, lymph node, and distant metastasis differ significantly between groups. Furthermore, we demonstrate that patients with non-metastatic pRCC had a significantly better cancer-specific mortality (HR 0.76, p = 0.007), when compared to ccRCC. Additionally, pRCC type 2 versus ccRCC exhibited no difference in cancer-specific mortality (HR 0.9, p = 0.722), whereas pRCC type 1 versus ccRCC displayed a risk of death reduced by 69% (p = 0.044). Taken together, outcome of pRCC versus ccRCC varies significantly in non-metastatic disease. Furthermore, pRCC type 2 exhibited no difference in cancer-specific mortality, whereas pRCC type 1 displayed a significantly reduced risk of death. Consequently, there is urgent need to respect histopathological entities and their subtypes, when assigning follow-up or targeted therapy to RCC patients.

## Introduction

Renal cell carcinoma (RCC) comprises a broad spectrum of malignancies, with indolent to very aggressive clinical behavior [1, 2]. Recently, the International Society of Urological Pathology (ISUP) added five new and three emerging RCC entities [3], further expanding the spectrum of renal tumors.

Papillary RCC (pRCC) is a heterogeneous disease with two morphologic groups (pRCC type 1 and 2) [4]. PRCC type 1 is a low-grade tumor with scanty basophilic cytoplasm, whereas type 2 displays high-grade features with pseudo-stratified tumor cell nuclei with bulky eosinophilic cytoplasm [5]. At ISUP conference there was agreement that pRCC subtyping is of value [3]. Some authors consider (oncocytic) pRCC type 3, which is, however, not generally accepted.

Furthermore, pRCC subtypes (type 1 and 2) were recently shown to be biologically distinct [6, 7]. Accordingly, RCC histopathology classification has been confirmed by (cyto)genetic analyses, with pRCC showing trisomy of chromosomes 7 and 17 and loss of chromosome Y, whereas clear cell RCC (ccRCC) frequently displays deletion of chromosome 3p and mutation of VHL gene [2]. Knowledge of these underlying molecular mechanisms has allowed for design of targeted agents such as tyrosine kinase or mTOR inhibitors, which, besides immunotherapy, are currently the hallmarks of systemic therapy in metastatic RCC [8].

Traditionally, pRCC is associated with a favorable prognosis as compared to ccRCC [9–12]. However, in other series, pRCC has equivalent or worse outcome than ccRCC [13–15]. Regarding pRCC subtype outcome, few studies contained inconsistent results [5, 7, 16–18]. Given this situation, our study will increase the understanding of oncological outcome of pRCC, pRCC subtypes and ccRCC and help to assign follow-up or targeted therapy for RCC patients.

## Patients and methods

### Design, patients, and pathologic evaluation

Clinical and pathological data of patients (n = 2,325) who underwent radical or partial nephrectomy for pRCC from 17 academic/ non-academic centers worldwide (14 European/ three North American centers, time period 1984–2015) were consecutively pooled in a retrospective database, whereby not all centers encompassed the entire period. In eight centers (seven European, one North American center; n = 962) subtyping of pRCC was performed. A distribution into the pRCC subtypes 1 and 2 was available since their inclusion in the WHO classification in 2004 (n = 547) [2]. For direct comparison with ccRCC, the retrospective Collaborative Research on Renal Neoplasms Association (CORONA) database was used, comprising 7,639 ccRCC from 11 academic/ non-academic centers (nine European, two US centers; 1984–2011). Patients under 18 years of age (n = 7) and patients with bilateral disease (n = 879) were excluded. Two centers comprising 1,374 patients and 12 patients from different centers were excluded due to inconclusive data. Additionally, 149 patients were excluded because of missing co-variables (stage or grade). Finally, clinical and pathological data of 7,543 patients (pRCC n = 1,943, ccRCC n = 5,600) were analyzed. In 429 cases, pRCC was classified in pRCC subtype 1 (n = 210) or 2 (n = 219). Eight cases with histopathological assessment of a combination of two subtypes were excluded in pRCC subtype analyses. Patients were separated in non-metastatic/ metastatic disease; patients with pathological lymph node invasion were assigned to the metastatic disease group. The reporting of the study has been conducted on the STROBE statement [19].

Surgical specimens were evaluated by genitourinary pathologists at each institution [1, 2], whereas no central pathology review was performed. Tumor stage was readjusted according to the TNM classification of malignant tumors of 2009 [20]. Preoperative staging of patients included abdominal CT or MRI, chest imaging, serum chemistry; bone scan and/ or brain imaging was performed when indicated by symptoms. No patient received (neo)adjuvant therapy. In metastatic disease or recurrence different therapeutic approaches in accordance with current guidelines were used. Cause of death was determined by treating physicians, chart review or death certificate. Approval of the study was obtained from the ethics committee of the State Medical Board Brandenburg, Germany, the ethics committee of the University Hospital Frankfurt, Goethe-University, Germany (#4/09), the local ethics committee of the Faculty Hospital Plzen and Faculty of Medicine Plzen, Charles University, Prague, Czech Republic, the Medical Ethics Committee II of the Faculty of Medicine Mannheim, University of Heidelberg, Germany (#2014-811R-MA), the ethics committee of the State Medical Board Westfalen-Lippe and the Faculty of Medicine University of Muenster, Germany (#2015-506-f-S), San Raffaele Ethics Committee, University Vita-Salute San Raffaele, Milan, Italy (#2007/29082007/V3), the ethical committee of the University of Heidelberg, Germany (#206/2005), the local human research ethics committee of the Medical Faculty of the University of Wuerzburg, Germany, the institutional review board of Weill Cornell Medical College, New York, NY, USA (#1007011131), UT Southwestern Institutional Review Board, Dallas, TX, USA, the Washington University in St. Louis Institutional Review Board, St. Louis, MO, USA (#201102423), the ethics committee of the Technical University of Dresden, Germany (EK 269072014), the ethics committee of the Medical University of Vienna and Vienna General Hospital, Austria, and the ethics committee of the Medical University of Graz, Austria. In most centers, written consent was obtained from the patients. In very few centers and permitted by the institutional review board/ local ethics committee, oral consent was obtained and documented in the patient chart.

### Outcome measurements and statistical analysis

Study end point was cancer-specific mortality (CSM). Group differences have been evaluated using t-tests for continuous and Chi^2-tests for nominal variables. For comparison of nominal variables between pRCC subtypes, exact Fisher tests with Monte Carlo computation of p-values were used. To illustrate CSM, cumulative incidence curves (CIC) and univariate Cox cause-specific hazard models were computed. To investigate the role of histological subtype as an independent prognostic factor, we used multivariate Cox models, adjusting for age as continuous variable and sex, Fuhrman grade and stage as nominal variables. A first multivariate analysis was used to assess the differences in survival between pRCC and ccRCC. To compare survival between pRCC subtypes and ccRCC, we used a second analysis including dummy variables to indicate the patient affiliation to one of four groups (ccRCC, pRCC, pRCC type 1/2). To examine the robustness of the results, sensitivity analyses were conducted, restricting either time frames or centers, which had been included in the main analysis. Finally, multi-collinearity between predictors was checked by calculating corresponding variance inflation factors.

A p-value <0.05 was considered significant. Descriptive and inferential statistics were performed as complete case analyses respective to prognostic factors of multivariate models. Statistical analysis was performed in R (version 3.2.3; R package ‘survival’) [21].

## Results

Survival of patients was calculated from time of renal surgery. Median follow-up was 64.6 months (95%CI 63.0–67.0), mean age of the patients was 61.8 years (range: 18.0–93.2). In non-metastatic disease, 5-year cumulative incidence rates (CIR) for dying of cancer for pRCC versus ccRCC and pRCC type 1 versus 2 were 6.4% versus 9.5% and 2.9% versus 8.0%. In metastatic disease, 5-year CIR for pRCC versus ccRCC was 64.9% versus 73.9%. Though comprising a relatively small series of patients, differences between the subgroups of pRCC were more apparent, with 5-year CIR for pRCC type 1 versus 2 of 20.9% and 68.0%. Clinical and pathological features of patients are summarized in Tables 1 and 2.

**Table 1:** Summary of clinical and pathological features and group differences between papillary renal cell carcinoma (pRCC) and clear cell renal cell carcinoma (ccRCC) (whole cohort, n = 7,543).

	pRCC		ccRCC		whole cohort		
	n	%	n	%	n	%	p
**Age at surgery**							0.94
<65 years	1,096	56.4	3,166	56.5	4,262	56.5	
≥65 years	847	43.6	2,434	43.5	3,281	43.5	
All	1,943	100.0	5,600	100.0	7,543	100.0	
**Sex**							<0.001
Female	474	24.4	2,243	40.0	2,717	36.0	
Male	1,469	75.6	3,357	60.0	4,826	64.0	
All	1,943	100.0	5,600	100.0	7,543	100.0	
**T classification**							<0.001
pT1a	862	44.4	1,854	33.1	2,716	36.0	
pT1b	433	22.3	1,347	24.1	1,780	23.6	
pT2a	163	8.4	397	7.1	560	7.4	
pT2b	82	4.2	134	2.4	216	2.9	
pT3a	288	14.8	1,125	20.1	1,413	18.7	
pT3b	75	3.9	604	10.8	679	9.0	
pT3c	8	0.4	36	0.6	44	0.6	
pT4	32	1.6	103	1.8	135	1.8	
All	1,943	100.0	5,600	100.0	7,543	100.0	
**Grade**							0.0021
G1	279	14.4	938	16.8	1,217	16.1	
G2	1,248	64.2	3,324	59.4	4,572	60.6	
G3	358	18.4	1,145	20.4	1,503	19.9	
G4	58	3.0	193	3.4	251	3.3	
All	1,943	100.0	5,600	100.0	7,543	100.0	
**Lymph node metastasis**							<0.001
pN0/pNx	1,800	92.6	5,390	96.2	7,190	95.3	
pN+	143	7.4	210	3.8	353	4.7	
All	1,943	100.0	5,600	100.0	7,543	100.0	
**Distant metastasis**							0.02
cM0	1,792	92.2	5,060	90.4	6,852	90.8	
cM1	151	7.8	540	9.6	691	9.2	
All	1,943	100.0	5,600	100.0	7,543	100.0	

**Table 2:** Summary of clinical and pathological features and group differences between pRCC subtypes (pRCC subtypes, n = 429).

	pRCC type 1		pRCC type 2		whole cohort		
	n	%	n	%	n	%	p
**Age at surgery**							0.06
<65 years	138	65.7	124	56.6	262	61.1	
≥65 years	72	34.3	95	43.4	167	38.9	
All	210	100.0	219	100.0	429	100.0	
**Sex**							0.49
Female	50	23.8	46	21.0	96	22.4	
Male	160	76.2	173	79.0	333	77.6	
All	210	100.0	219	100.0	429	100.0	
**T classification**							<0.001
pT1a	118	56.2	74	33.8	192	44.8	
pT1b	46	21.9	53	24.2	99	23.1	
pT2a	18	8.6	15	6.8	33	7.7	
pT2b	6	2.9	4	1.8	10	2.3	
pT3a	21	10.0	52	23.7	73	17.0	
pT3b	0	0.0	15	6.8	15	3.5	
pT3c	0	0.0	2	0.9	2	0.5	
pT4	1	0.5	4	1.8	5	1.2	
All	210	100.0	219	100.0	429	100.0	
**Grade**							<0.001
G1	46	21.9	9	4.1	55	12.8	
G2	140	66.7	150	68.5	290	67.6	
G3	18	8.6	49	22.4	67	15.6	
G4	6	2.9	11	5.0	17	4.0	
All	210	100.0	219	100.0	429	100.0	
**Lymph node metastasis**							0.01
pN0/pNx	202	96.2	197	90.0	399	93.0	
pN+	8	3.8	22	10.1	30	7.0	
All	210	100.0	219	100.0	429	100.0	
**Distant metastasis**							<0.001
cM0	201	95.7	189	86.3	390	90.9	
cM1	9	4.3	30	13.7	39	9.1	
All	210	100.0	219	100.0	429	100.0	

The pRCC cohort exhibited more male patients than ccRCC cohort, presented with lower tumor stages at diagnosis and exhibited more patients with regional lymph node metastasis, but fewer patients with distant metastasis (Table 1). Patients with pRCC type 2 exhibited higher stage and grade and more regional lymph node/ distant metastases than patients with type 1 (Table 2).

**Fig 1:**
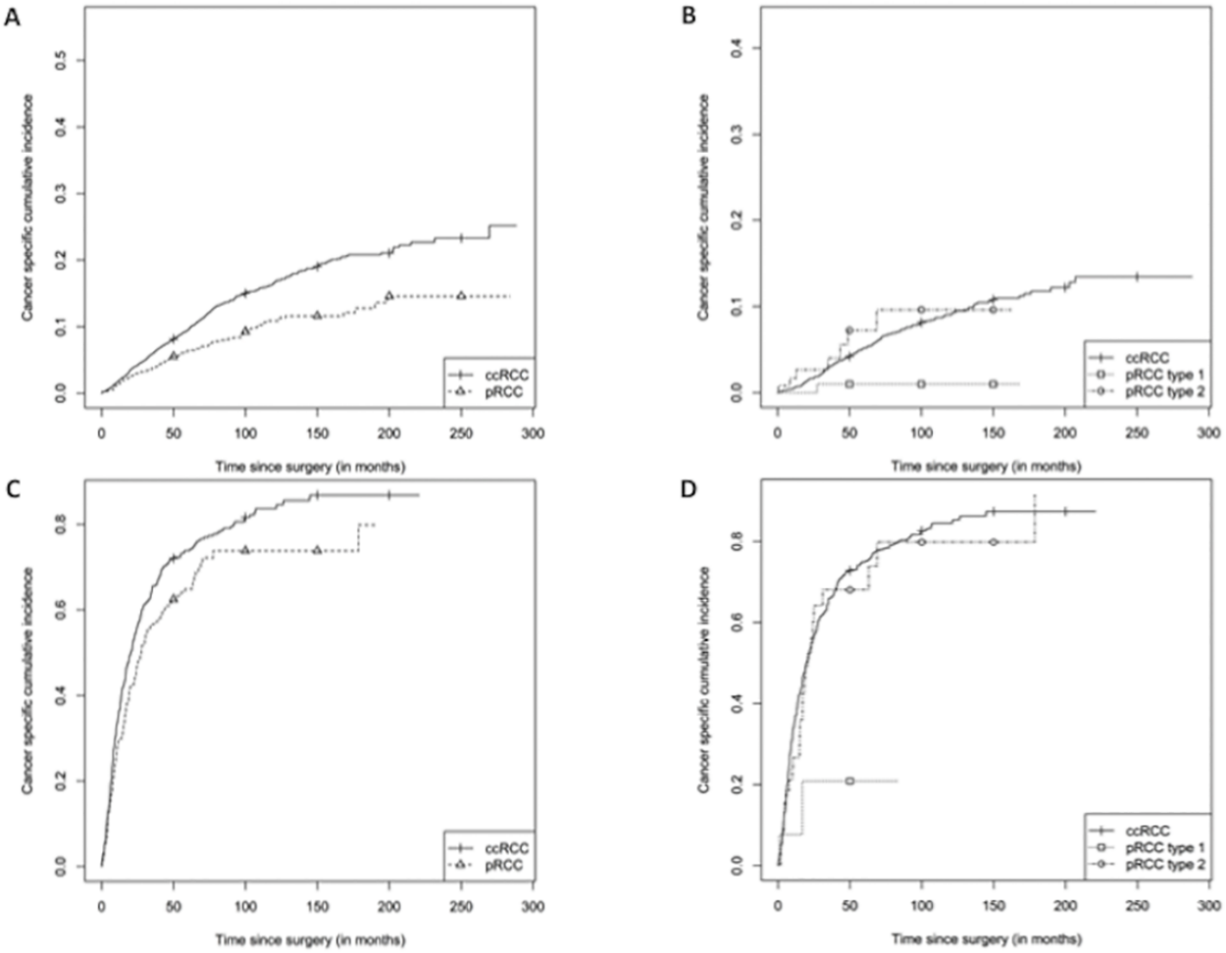
Cumulative incidence curves for cancer-specific mortality. A) Non-metastatic disease, papillary renal cell carcinoma (pRCC) versus clear cell renal cell carcinoma (ccRCC); B) Non-metastatic disease, pRCC subtypes (type 1 and type 2) versus ccRCC; C) Metastatic disease, pRCC versus ccRCC; D) Metastatic disease, pRCC subtypes (type 1 and type 2) versus ccRCC.

CIC and univariate survival analysis are depicted in Fig 1 and Table 3.

**Table 3:** Uni- and multivariate Cox cause-specific hazards analysis of pRCC, ccRCC, and pRCC subtypes (type 1 and type 2) and clinical/ pathological variables for the prediction of cancer-specific mortality in patients with non-metastatic and metastatic RCC.

	Univariate analysis			Multivariate analysis		
	HR	95%CI	p	HR	95%CI	p
**Patients with non-metastatic disease (M0)**						
Age	1.02	1.01–1.03	<0.001	1.02*	1.01–1.03	<0.001
Sex (Female vs. Male)	0.74	0.63–0.87	<0.001	0.73*	0.62–0.86	<0.001
pT (pT 3–4 vs. pT 1–2)	4.16	3.57–4.85	<0.001	3.19*	2.73–3.74	<0.001
Grade (G 3–4 vs. G 1–2)	4.78	4.1–5.57	<0.001	3.75*	3.21–4.38	<0.001
pRCC vs. ccRCC	0.61	0.5–0.75	<0.001	0.76	0.62–0.93	0.007
pRCC type 2 vs. type1	4.59	1.31–16.11	0.017	2.9	0.83–10.19	0.097
pRCC type 1 vs. ccRCC	0.2	0.06–0.62	0.005	0.31	0.1–0.97	0.044
pRCC type 2 vs. ccRCC	0.91	0.52–1.57	0.722	0.9	0.52–1.57	0.722
**Patients with metastatic disease (M1)**						
Age	1	0.99–1.01	0.753	1*	1–1.01	0.304
Sex (Female vs. Male)	0.9	0.75–1.08	0.266	0.87*	0.72–1.05	0.135
pT (pT 3–4 vs. pT 1–2)	1.44	1.2–1.73	<0.001	1.4*	1.16–1.69	<0.001
Grade (G 3–4 vs. G 1–2)	1.49	1.26–1.77	<0.001	1.46*	1.22–1.73	<0.001
pRCC vs. ccRCC	0.85	0.7–1.04	0.108	0.82	0.67–1	0.05
pRCC type 2 vs. type1	4.39	1.04–18.61	0.045	3.51	0.83–14.85	0.088
pRCC type 1 vs. ccRCC	0.24	0.06–0.97	0.046	0.29	0.07–1.16	0.079
pRCC type 2 vs. ccRCC	1.02	0.68–1.52	0.933	1.01	0.67–1.52	0.957

* HRs are based on two different Cox models and therefore might slightly differ

Multivariate Cox analyses (Table 3) revealed that patients suffering from non-metastatic pRCC significantly correlated with reduced risk of cancer specific death (HR 0.76, p = 0.007) compared to ccRCC. Apart from pRCC, age, sex, stage, grade and pRCC type 1 were identified as independent predictors. PRCC type 1 (versus ccRCC) displayed a risk of death reduced by 69% (p = 0.044). PRCC type 2 (versus 1) displayed a HR of 2.9, indicating a clear coherence despite a non-significant p-value. Nearly no difference in CSM between pRCC type 2 and ccRCC (HR 0.9, p = 0.722) was demonstrated. We found our results to be robust when we excluded the time frame before pRCC subtyping was included in the WHO classification and centers not differentiating between pRCC subtypes.

In metastatic disease, only stage and grade were identified as independent predictors of CSM. PRCC and pRCC type 1 versus ccRCC displayed a reduced risk of death by 18% and 71%, whereas pRCC type 2 versus 1 revealed a 3.51 times greater risk, though no statistical significance was reached. Similar to non-metastatic disease, no difference in CSM between metastatic pRCC type 2 and ccRCC was observed. Furthermore, when the analyses were restricted to the post tyrosine kinase inhibitor era, pRCC type 2 versus 1 revealed a 4.63 times greater risk of death (p = 0.044) (Table 4).

**Table 4:** Uni- and multivariate Cox cause-specific hazards analysis of pRCC, ccRCC, and pRCC subtypes (type 1 and type 2) and clinical and pathological variables for the prediction of cancer-specific mortality in patients with metastatic RCC in the post tyrosine kinase inhibitor era.

	Univariate analysis			Multivariate analysis		
	HR	95%CI	p	HR	95%CI	p
**Patients with metastatic disease (M1)**						
Age	1	0.98–1.02	0.916	1*	0.98–1.02	0.891
Sex (Female vs. Male)	1.06	0.67–1.7	0.796	1.06*	0.66–1.71	0.815
pT (pT 3–4 vs. pT 1–2)	1.31	0.84–2.04	0.237	1.21*	0.76–1.92	0.421
Grade (G 3–4 vs. G 1–2)	1.41	0.91–2.2	0.125	1.36*	0.86–2.16	0.187
pRCC vs. ccRCC	0.89	0.58–1.36	0.579	0.86	0.56–1.34	0.514
pRCC type 2 vs. type1	5.12	1.17–22.31	0.03	4.63	1.05–20.52	0.044
pRCC type 1 vs. ccRCC	0.24	0.06–1.02	0.053	0.29	0.07–1.21	0.09
pRCC type 2 vs. ccRCC	1.27	0.7–2.27	0.431	1.34	0.74–2.42	0.33

* HRs are based on two different Cox models and therefore might slightly differ

Finally, variance inflation factors for all covariates were <1.1. Hence, any relevant collinearity effects can be ruled out.

## Discussion

We comparatively evaluated the outcome of pRCC versus ccRCC in two large multi-institutional databases, including distribution of pRCC subtypes 1 and 2. Our findings concerning CSM rates and group differences agree with previous publications. Studies differentiating metastatic and non-metastatic disease displayed longer 5-year CSS rates for pRCC versus ccRCC in non-metastatic disease [14, 22, 23]. In metastatic disease, information differs: where our study showed better CSM rates for pRCC than ccRCC, other studies displayed equal [14] or worse [22] CSS rates in pRCC. In metastatic pRCC subtypes, probably due to low patient numbers, no 5-year CSM rates are available. Consequently, we are the first group to present such data. Equal to our results, former studies revealed that pRCC (versus ccRCC) displayed more male patients and lower tumor stages [9, 10, 12–15, 22–24]. Our results that pRCC harbor more regional lymph node metastasis but less distant metastasis at time of diagnosis are in accordance with previous studies [9, 10, 12, 13, 22–24]. Again similar to our data, patients with pRCC type 2 presented with higher tumor stages, higher grades, more regional lymph node metastases, and more distant metastases [4, 5, 7, 16, 23, 25, 26], compared to patients with pRCC type 1. In conclusion, the results are most likely determined by the underlying tumor biology.

Substantially less consistent is the situation regarding outcome of pRCC: our results are in line with the traditional hypothesis that pRCC is associated with a favorable prognosis compared to ccRCC. In the largest study published to date, 2,278 pRCC patients, 13,841 ccRCC patients and 1,486 patients with other RCC entities showed histopathology significantly associated with CSS on multivariate analysis. Here, patients with pRCC histology had improved survival (HR 0.85) compared to ccRCC [9]. Similarly, in a relatively large multi-institutional study, demonstrating histopathology as a predictor of CSM in multivariate analysis, pRCC displayed more favorably (HR 0.7, p = 0.024) than ccRCC [11]. Additionally, two smaller single institution series reported significant differences in outcome between ccRCC compared to pRCC/ chromophobe RCC (chRCC) [10] or revealed histopathology as an independent predictor of metastasis/ death [12]. However, in other series, pRCC was reported to have equivalent or worse prognosis than ccRCC. In a relatively large multi-institutional study comprising pRCC (n = 396), chRCC (n = 103), and ccRCC (n = 3,564) with a median follow-up of 43 months, TNM classification, Fuhrman grade, and ECOG performance score, but not histopathology were retained as independent prognostic variables in multivariate analysis [14]. Accordingly, two single US institution series did not reveal histopathology as independent marker of CSS [13] or differences between pRCC and ccRCC [15]. These studies reported a shorter follow-up and/ or less patients studied than our study. In a large subgroup of patients with RCC and tumor thrombus who underwent tumor thrombectomy, pRCC was independently associated with poor CSS in multivariate analysis (HR 1.62, p<0.05) [27]. Additionally, in metastatic non-clear cell disease, including pRCC, outcome of patients under systemic therapy is worse than in patients with ccRCC [28]. Analogous to the latter two studies, Steffens et al. compared retrospective data of patients with pRCC (n = 565) to ccRCC (n = 4,376) with a median follow-up of 46.7 months [22]. Multivariate analysis revealed pRCC as significant favorable prognostic factor in localized disease (HR 0.45), but as a negative prognostic factor in metastatic (HR 1.37) disease. Therefore, the question was raised whether these groups correspond to the pRCC subtypes 1 and 2 or are characterized by other alterations. Finally, we cannot support their result of an unfavorable outcome of metastatic pRCC, since in our metastatic pRCC cohort, with a distribution of 76.9% being pRCC type 2 and 23.1% being type 1, pRCC and pRCC type 1 versus ccRCC displayed a reduced risk of death, whereas no difference in CSM between metastatic pRCC type 2 and ccRCC was observed. Therefore, it remains unclear why the outcome of pRCC versus ccRCC varies between non-metastatic and metastatic disease in their study, whereby the results are supported by real-life scenario on metastatic non-ccRCC outcome [28]. Another study revealed histopathology as independent predictor of CSS (p = 0.03) in a multivariate model, however little gain (+0.1%) in predictive accuracy was seen. Therefore, the authors concluded that, from a statistical perspective, the various histopathologies might be included as single entity [24]. Although speculative, the predictive accuracy would be greater, had it been possible to differentiate the included pRCC in subtypes 1 and 2.

Few other studies published contain inconsistent results regarding pRCC subtype outcome with a maximum of 486 pRCC patients. Statistical limitations or shorter follow-up were often present when compared to our work. Two studies retained pRCC subtype as independent prognostic factor on multivariate analysis [5, 16], three others exhibited pRCC subtype not significantly associated with CSS in multivariate analysis [7, 17, 26]. The largest pRCC subtype series to date (type 1 n = 369, type 2 n = 117), comprising only patients treated with nephron-sparing surgery for T1-3 tumors with a mean follow-up of 35 months in a retrospective multi-institutional setting, displayed patients with pRCC type 1 with an equal risk of RCC death compared to type 2 (HR 0.9, p = 0.89) [26]. In our slightly smaller patient cohort, comprising 429 non-metastatic and metastatic pRCC subtypes with a considerably longer follow-up, non-metastatic pRCC type 1 (vs. ccRCC) remained as independent favorable prognostic factor of CSM in multivariate analysis, whereas non-metastatic pRCC type 2 exhibited no difference in CSM. Consequently, our results clearly support the ISUP conference consensus, that tumors should be classified as type 1 and 2.

Despite academic interest, outcome of pRCC versus ccRCC matters in daily clinical practice. In non-metastatic disease, our work regards follow-up decisions: patients with pRCC type 1 should obtain a reduced follow-up scheme compared to patients with ccRCC or pRCC type 2. In metastatic disease, our data can help when assigning systemic therapy: Whereas the EAU guideline [8] discriminates between ccRCC and non-ccRCC, based on our data, a patient with a metastatic pRCC type 2 has a nearly equal risk of dying from this disease as a patient with a metastatic ccRCC. Consequently, this patient needs a therapy with equal efficacy as for ccRCC. On the other hand, a patient with a pRCC type 1 might need active surveillance with delayed systemic therapy. Given the molecular differences of pRCC, their subtypes, and ccRCC, targeted agents such as tyrosine kinase inhibitors show significantly less efficacy in tumors with non-ccRCC including pRCC histology [28, 29]. Given mutations of the mesenchymal-epithelial transition (MET) oncogene in pRCC type 1 and of fumarate hydratase (FH) in pRCC type 2 [2], targeted agents assigning MET (e.g. the recently FDA approved tyrosine kinase inhibitor cabozantinib targeting MET, VEGFR, and AXL [30]) or FH have probably higher efficacy in these patients. Despite the underlying tumor biology, which defines future targeted therapies, an accurate assignment of already approved agents is also of importance. Recently, the randomized phase 2 ASPEN trial showed a longer median progression-free survival in pRCC patients treated with sunitinib compared to everolimus [31], further corroborating the need for a more diversified histopathology-based strategy in assigning targeted agents. Additionally, this also further underlines the necessity to obtain a renal tumor biopsy prior to definitive treatment of metastatic RCC, in patients in whom cytoreductive nephrectomy is not being performed or a former histopathology has been inconclusive.

Several limitations of our study should be noted: First, our work is based on retrospective data and a central pathology review was not performed. Although re-classification of tumors according to the ISUP criteria [3] possibly minimizes the risk of misclassification, central pathology review can hardly be performed in 7,543 cases from two worldwide databases. Second, discrimination between pRCC subtype 1 and 2 was performed in eight of 17 centers, therefore, corresponding data were available in 429 cases. This situation reflects the real-world scenario of pathologists, where only 59% of the respondents of the ISUP preconference survey noted that they classified tumors according to subtypes 1 and 2 [3]. Third, variables such as concomitant sarcomatoid differentiation or tumor necrosis were recorded in both databases, but due to missing values of 20–30% these were not selected for further analysis, otherwise displaying a maximum of 1.5% missing values. However, to date, this is the largest international multi-institutional study of non-metastatic and metastatic pRCC, pRCC type 1 and 2, and ccRCC with a long median follow-up.

## Conclusions

Patients with non-metastatic pRCC showed a significantly reduced risk of cancer specific death, when compared to ccRCC. Additionally, pRCC type 1 displayed a risk of death reduced by 69%, whereas pRCC type 2 exhibited no difference in CSM, which might indicate an analogous clinical behavior of these two RCC entities. There is urgent need to consider histopathological entities and their subtypes, when assigning follow-up or targeted therapy for RCC patients.

## Supporting information

S1 Table Dataset. (XLSX)
